# Integrative approaches to depression in end-stage renal disease: insights into mechanisms, impacts, and pharmacological strategies

**DOI:** 10.3389/fphar.2025.1559038

**Published:** 2025-04-14

**Authors:** Cheng Yuan, Fengpei Chang, Hongfu Zhai, Jiayin Du, Danqin Lu, Haoli Ma, Xiaoyan Wu, Ping Gao, Lihua Ni

**Affiliations:** ^1^ Department of Oncology, Yichang Central People's Hospital, The First College of Clinical Medical Science, China Three Gorges University, Yichang, Hubei, China; ^2^ Department of Nephrology, Xijing Hospital, Fourth Military Medical University, Xi'an, Shaanxi, China; ^3^ Department of Nephrology, Zhongnan Hospital, Wuhan University, Wuhan, China; ^4^ Department of Biological Repositories, Zhongnan Hospital of Wuhan University, Wuhan, China; ^5^ Department of General Practice, Zhongnan Hospital of Wuhan University, Wuhan, China

**Keywords:** depression, mortality, end stage renal disease, renal function, hemodialysis

## Abstract

Depression is a frequently overlooked psychiatric symptom in patients with end-stage renal disease (ESRD), seriously affecting their quality of life, risk of death, adherence to treatment, cognitive abilities, and overall health outcomes. The study investigates the prevalence of depression is in ESRD patients, along with the methods for assessment, diagnostic guidelines, underlying factors, consequences, and management strategies. The Beck Depression Inventory (BDI), with an optimal diagnostic cutoff score greater than 14, has been identified as the most accurate for diagnosing depression in ESRD, while emerging tools such as vacancy-driven high-performance metabolic assays show promise for evaluation. Depression contributes to adverse health outcomes by increasing risks of treatment withdrawal, suicide, and cognitive impairment, as well as serving as a predictor of mortality and poor treatment adherence. Even though tricyclic antidepressants and selective serotonin reuptake inhibitors are commonly used, the effectiveness of treatment remains unpredictable because clinical studies often have limitations such as small sample sizes, no randomization, and missing control groups. Innovative approaches, such as nanomaterials and traditional Chinese medicine, have shown therapeutic potential with reduced side effects. Future research should focus on specific high-risk populations, particularly older adults and women under the age of 45, to better tailor interventions. The goal of this research is to improve understanding of depression in ESRD, leading to better patient care, improved quality of life, and superior clinical results.

## Main contents

• **Prevalence**: Depression is a common yet often underrecognized symptom in individuals with end-stage renal disease (ESRD). Its prevalence varies across different stages of renal treatment, with patients on hemodialysis experiencing higher rates of depression than those on peritoneal dialysis (PD).
• **Assessment and Diagnosis**: The evaluation of depression in patients with ESRD has been extensively studied. Among various assessment tools, the Beck Depression Inventory (BDI), with an optimal diagnostic cutoff score greater than 14, has demonstrated the highest accuracy. Recently, a novel approach utilizing a vacancy-engineered cobalt oxide (Vo-Co_3_O_4_) assisted laser desorption/ionization mass spectrometry platform has been introduced for the diagnosis and therapeutic evaluation of depression.
• **Mechanism**: The etiology of depression in ESRD is multifaceted, as patients frequently present with numerous comorbidities. Greater emphasis should be placed on investigating the kidney-brain axis and the role of uremic toxins.
• **Influence**: Depression adversely impacts health outcomes, elevates the risks of withdrawal and suicide, contributes to cognitive impairment, predicts mortality and sarcopenia, and results in non-adherence among patients with ESRD.
• **Strategies**: Managing depression in ESRD patients is challenging due to altered drug metabolism and limited antidepressant safety data. Selective serotonin reuptake inhibitors (SSRIs) are the first choice for treatment. Promising future options include the use of nanomaterials and traditional Chinese medicine. Non-drug methods, such as exercise and cognitive behavioral therapy, are essential. Future research should focus on safer antidepressants and robust clinical trials to improve treatment.
• **Special population**: Greater attention should be directed towards older adults and women under 45 with ESRD.

## 1 Depression and its impact on end-stage renal disease patients

Depression is widely recognized as a complex comorbid condition associated with various chronic diseases, including renal disease, cancer, diabetes, digestive disorders, and obesity. The occurrence of depression in these groups has been associated with hindered recovery and higher death rates. For patients undergoing maintenance hemodialysis (HD), the survival rates are 83.0% at 1 year, 78% at 2 years, 69% at 3 years, and 57% at 5 years (Chi et al., 2017). These statistics highlight the importance of addressing the psychological health of individuals with end-stage renal disease (ESRD), particularly in light of the rising prevalence and essential nature of dialysis therapies.

The etiology of depression in ESRD is multifactorial and complex. Patients with ESRD face both physical and psychological burdens that contribute to the development and exacerbation of depression. Physiologically, the progression of kidney disease leads to the accumulation of uremic toxins and increased inflammatory cytokines, such as interleukin-6 (IL-6), which negatively affect brain function and mood regulation, thereby linking kidney disease to depressive symptoms (Jayakumar et al., 2023; Yuan et al., 2021; Bugnicourt et al., 2013; Liu et al., 2024a). Psychologically, ESRD patients face significant challenges, including dialysis, frequent hospitalizations, declining kidney function, and physical limitations, all of which contribute to emotional distress, helplessness, and depression (Natale et al., 2019a). Reduced life expectancy and economic burden further exacerbate these mental health risks, highlighting the need for research into their psychological wellbeing (Halen et al., 2012; Yang et al., 2020).

A two-decade bidirectional cohort study found a reciprocal link between depression and chronic kidney disease (CKD): depressive symptoms increased the risk of CKD (Liu et al., 2022), and impaired kidney function raised the risk of depression. This highlights the need for careful management of both conditions to prevent progression. Since both are modifiable risk factors, early interventions by healthcare providers are crucial. Additionally, depression predicts mortality in ESRD patients, necessitating further research to understand its impact on disease progression and survival, with the goal of enhancing both psychological and clinical outcomes in this group.

This review aims to provide a comprehensive synthesis of the current understanding of depression in patients with ESRD, focusing on its prevalence, underlying factors, and management strategies. A thorough literature search was conducted across major databases, including PubMed, Scopus, and Google Scholar. The search focused on studies relevant to depression in ESRD patients, with no restrictions on publication date. Studies were included if they (i) examined depression in adult ESRD patients, (ii) provided data on depression prevalence, diagnostic methods, or underlying factors, and (iii) discussed treatment strategies or management approaches for depression in this population. Studies were excluded if (i) they were not published in English, (ii) focused on other psychiatric conditions. This review is based on a synthesis of these selected studies to provide a comprehensive overview of depression in ESRD, its consequences, and potential therapeutic interventions.

## 2 Prevalence of depression in pre-dialysis, dialysis, and peritoneal dialysis (PD)

Research has shown varying prevalence rates of depression among patients with pre-dialysis CKD, HD, and peritoneal dialysis (PD) populations (Table 1). In eastern Turkey, 35.0% of 120 CKD patients not undergoing dialysis had depression scores exceeding the threshold, according to validated diagnostic tools (Cantekin et al., 2014). Similarly, a Chinese study involving 334 CKD patients, 77.5% of whom were in stages 1–3, found a depression prevalence of 22.2%, highlighting the presence of psychopathological issues even before dialysis initiation (Duan et al., 2021). Higher rates of depression have been documented in patients undergoing dialysis. A cross-sectional study in Lebanon, utilizing the Hospital Anxiety and Depression Scale (HADS), reported a depression prevalence of 40.8% among 83 HD patients at a tertiary medical center (Semaan et al., 2018). In Brazil, an investigation using the Beck Depression Inventory II (BDI-II) in 148 HD patients revealed a depression prevalence of 68.2%, with severity classified as mild (49.5%), moderate (41.5%), and severe (9%) (Silva Junior et al., 2014). Klein et al. (1981) estimated a 15% depression rate in ESRD patients, while other studies demonstrated that at least 25% of patients on HD and 30% of those on PD exhibit depressive symptoms (Juergensen et al., 1996; Kimmel et al., 1993; Kimmel et al., 1995). Foster et al. (1973) and Smith et al. (1985) independently reported a 47% prevalence of depression among ESRD and HD populations. Remarkably, Reichsman and Levy (1972) observed a 100% prevalence of depression in their HD cohort, further underscoring the widespread burden of this condition.

**TABLE 1:** Prevalence of depression in pre-dialysis, dialysis, and peritoneal dialysis.

Disease	Pre-dialysis	Dialysis	Peritoneal dialysis (PD)
Prevalence of depression	22.2% (6)	35.0% (5)	30% (10)
		40.8% (7)	20.4% (17)
		68.2% (9)	
		100 (15)	

The association between depression and dialysis modality remains a topic of debate. Some studies indicate no significant difference in depression levels between HD and PD patients (He et al., 2021). However, other research suggests that patients receiving HD may experience a higher prevalence of depression compared to those on PD (30.6% *versus* 20.4%) (Adejumo et al., 2024). Further exploration is required to clarify the potential effects of dialysis modality on the mental wellbeing of ESRD patients, given these conflicting results.

## 3 Identification and measurements of depression in ESRD

Depression is often underrecognized and underestimated in patients with ESRD, primarily due to three factors. First, the symptomatology of depression frequently overlaps with uremic symptoms associated with ESRD. Individuals afflicted by both conditions commonly report experiencing fatigue, apathy, impaired concentration, motor tremors, reduced appetite, gastrointestinal disturbances, and sleep disorders. These symptoms are characteristic of both uremia and depression, complicating early diagnosis of depression and contributing to variability in its reported prevalence. Second, patients often deny their illness, potentially minimizing or obscuring symptoms, which challenges the accurate assessment of depression. Lastly, variability in the prevalence of depression is linked to various methodological issues. Therefore, accurate and reliable measures are essential to differentiate depressive symptoms from uremic symptoms in ESRD patients.

Among the available diagnostic tools, the Beck Depression Inventory (BDI) has demonstrated high accuracy in this population, with an optimal diagnostic cutoff score greater than 14. Depression severity is categorized as follows: 0–13 (Minimal), 14–19 (Mild), 20–28 (Moderate), and 29–63 (Severe). Table 2 summarizes common assessment tools used for diagnosing depression in ESRD. In addition to the BDI, significant advancements in diagnostic methods have been made. Recent evidence suggests that multiple assessments of depression over time may provide a more accurate prediction of mortality compared to single evaluations. For example, proton magnetic resonance spectroscopy (^1^H-MRS) has shown promise in detecting metabolite changes in the brains of ESRD patients with depression, offering a foundation for early diagnosis and assessment of depression severity in this population (Wang et al., 2020). This underscores the urgent need for early and effective diagnostic tools for depression in ESRD.

**TABLE 2:** Tools for assessing depression in ESRD.

Tools	References
Beck Depression Inventory-II (BDI-II)	Nadort et al. (2022)
Cognitive depression index (CDI)	Vučković et al. (2023)
Hamilton rating scale for depression (HAM-D)	Chen et al. (2022)
Zung Self-Rating Depression Scale	Butt et al. (2022)
SF-36 Health Survey	Xiong et al. (2022)
Diagnostic interview schedule (DIS)	Raine et al. (2022)
Structured Clinical Interview for the DSM (Diagnostic and Statistical Manual of Mental Disorders)-IV (SCID-I)	Widing et al. (2022)
Kidney Disease Quality of Life Questionnaire (KDQOL-SF)	Dam et al. (2022)
Patient Health Questionnaire-9 (PHQ-9)	Santos et al. (2022)
Center for Epidemiologic Studies Depression Scale (CES-D)	Chiou et al. (2023)
Proton magnetic resonance spectroscopy	Poudel et al., 2022; Wang et al., 2020
Vacancy-Driven High-Performance Metabolic Assay	Chen et al. (2024)
Conversational AI bot	Kaywan et al. (2023)

AI, Artificial intelligence.

Beyond traditional methods, new diagnostic technologies are being investigated. Chen et al. (2024) developed a high-performance metabolite-based test using vacancy-engineered cobalt oxide (Vo-Co_3_O_4_) with laser desorption/ionization mass spectrometry. These optimized nanoparticles enhance signals and allow for strong plasma metabolic fingerprint recordings. This progress contributes to the development of diagnostic tools for assessing depression and provides new insights for its management in ESRD patients.

Artificial intelligence (AI) has also become a promising tool in interventions for behavioral health. Payam and colleagues developed a chatbot for depression analysis, using Dialogflow as the conversational platform. This comprehensive mass screening method can detect early depression and assist in facilitating automated and nuanced communication. Although it is not meant to substitute mental health experts, the chatbot shows promise in improving early detection using validated scoring methods. Collectively, these cutting-edge diagnostic tools and technologies offer potential for enhancing the early detection and treatment of depression in patients with ESRD.

An effective team is crucial for evaluating and treating depression in ESRD patients, ensuring precise assessments and successful management. When the diagnosis of depression is unclear, it is recommended to consult a mental health professional before starting any medication. Not every ESRD patient suspected of having depression requires a referral to a psychiatrist. A comprehensive depression screening should be conducted first. Tools like the BDI, Patient Health Questionnaire (PHQ-9), and Center for Epidemiological Studies Depression Scale (CES-D) are frequently used for screening. Diagnosing depression in uremic patients is challenging due to overlapping symptoms. Self-reported scales like the BDI may lead to overdiagnosis by placing too much on physical symptoms (Hedayati et al., 2006). In HD patients, the BDI (cutoff ≥14) showed 62% sensitivity and 81% specificity, while the PHQ-9 (cutoff ≥10) had the best performance with 92% sensitivity and specificity. The CES-D (cutoff ≥18) had 69% sensitivity and 83% specificity (King-Wing Ma and Kam-Tao Li, 2016). These results highlight the need for careful selection and integration of screening tools in a multidisciplinary approach for managing depression in ESRD patients.

## 4 Mechanism of depression in ESRD

**FIGURE 1:**
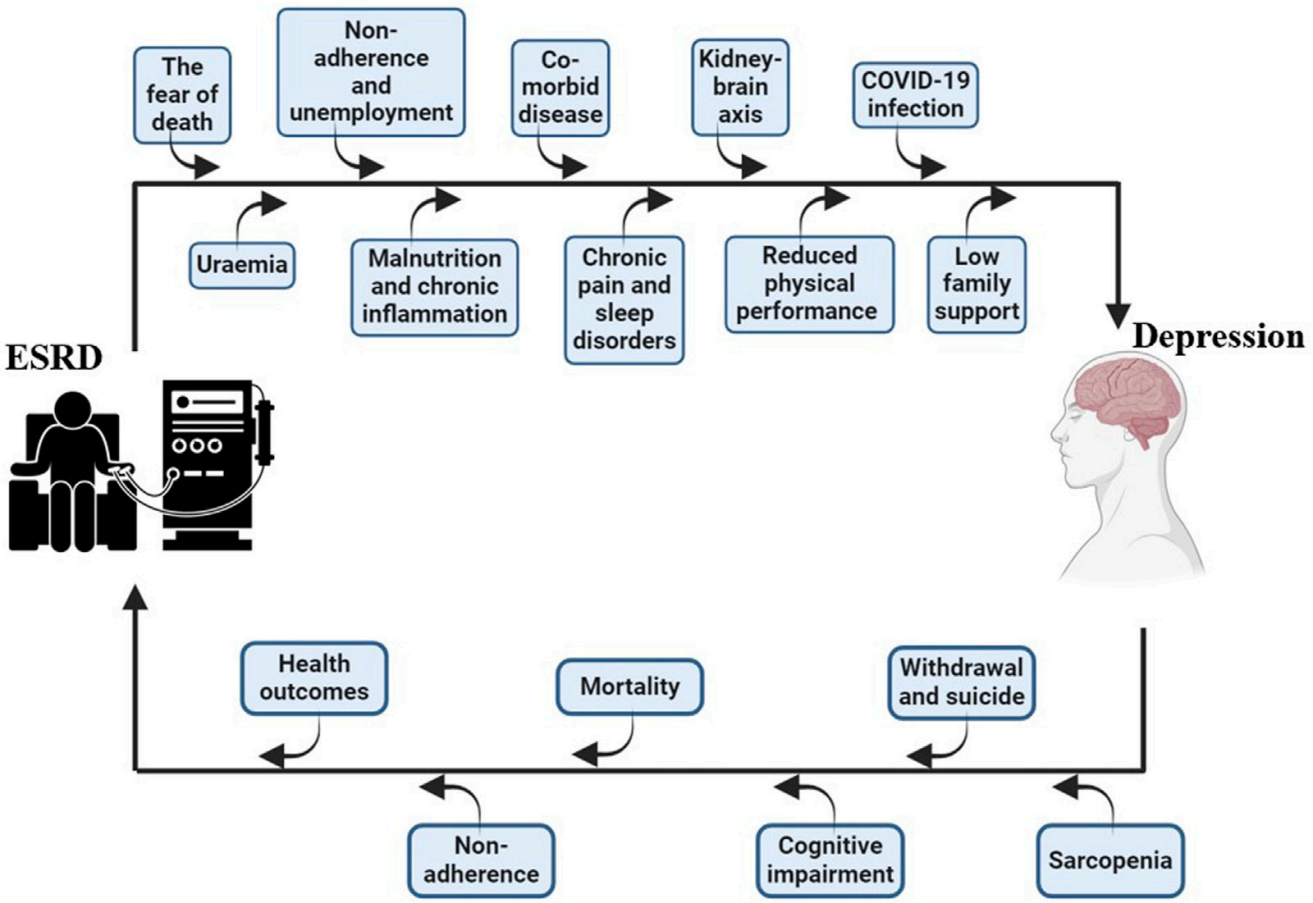
The crosstalk between ESRD and depression.

The mechanism of depression in patients with ESRD is multifactorial, as these individuals often face numerous comorbid conditions (Gerogianni et al., 2018). The contributing factors can be summarized in Figure 1 as follows: 1) Comorbid Diseases: Depression in ESRD is frequently influenced by coexisting conditions, such as cardiovascular disease (CVD), diabetes, and immune dysfunction (Fan et al., 2014); 2) Malnutrition and Chronic Inflammation: These factors are prevalent in ESRD patients and significantly contribute to depressive disorders (Guenzani et al., 2019; Choi et al., 2012); 3) Chronic Pain and Sleep Disorders: Persistent pain and disrupted sleep are common and associated with high rates of depression in ESRD (Liu et al., 2021; Pandi-Perumal et al., 2020); 4) Non-Adherence: Non-adherence to treatment regimens is both a consequence and a contributing factor to depression (Gebrie and Ford, 2019); 5) Uremia: The accumulation of uremic toxins negatively impacts the central nervous system, exacerbating depressive symptoms; 6) Decreased Sexual Function: Impaired sexual health is a significant psychological burden for many ESRD patients; 7) Fear of Death: The existential threat posed by ESRD and its life-sustaining treatments can lead to profound anxiety and depression; 8) Low Family Support: Limited familial support may impair patients’ self-care abilities and worsen their emotional wellbeing; 9) Reduced Physical Performance: Decreased motivation and physical limitations further contribute to depressive symptoms; 10) COVID-19 and the Kidney-Brain Axis: The recent COVID-19 pandemic has added an additional layer of stress, potentially affecting the kidney-brain axis and exacerbating depression in ESRD patients.

Among these factors, the impact of COVID-19 on depression is particularly noteworthy. Patients with ESRD are more vulnerable to COVID-19 infection due to their compromised immune systems and the high-risk environment of HD centers during viral outbreaks. Studies have shown that the prevalence of depression in patients with COVID-19 can be as high as 45% (Deng et al., 2021). Moreover, depression and stress induced by COVID-19 can disrupt the immune system, exacerbating the severity of the infection (2021). The mechanisms underlying COVID-19-induced depression include elevated levels of pro-inflammatory cytokines, increased cortisol levels, mitochondrial dysfunction, and deficiencies in vitamin D3 and nutrition (Mohammadkhanizadeh and Nikbakht, 2021; Mazza et al., 2020). Additionally, ESRD patients with comorbidities are at heightened risk of mortality from COVID-19. A comprehensive understanding of the mechanisms and factors contributing to depression in COVID-19 patients is crucial for developing effective therapeutic strategies, which may help enhance preparedness for future pandemics, especially among vulnerable populations such as HD patients.

The kidney-brain axis has garnered attention due to shared features between the brain and kidneys, linking CKD-related issues like white matter injury and vascular dysfunction to neurodegenerative diseases such as depression (Ariton et al., 2021; Xie et al., 2022; Miranda et al., 2017). Altered cerebral hemodynamics in CKD patients is tied to cognitive impairment. Understanding these interactions is crucial for creating targeted treatments for depression in CKD patients (Viggiano et al., 2020; Kim et al., 2022).

Additionally, uremic toxins from renal failure contribute to depression in ESRD (Duranton et al., 2012), as PD and HD only partially remove these toxins, which cause oxidative stress and inflammation, affecting the central nervous system (CNS) and disrupting neurotransmitter function (Miller and Raison, 2016). Furthermore, depression is strongly associated with stress-related mechanisms, including the hypothalamic-pituitary-adrenal (HPA) axis, which can be greatly affected by the composition of the gut microbiome (Sudo et al., 2004). Imbalances in gut microbiota play a role in depression by affecting systemic inflammation, disrupting the HPA axis, and changing neurotransmitter signaling (Peirce and Alviña, 2019).

Depression in ESRD can also be conceptualized within the framework of perceived loss. This includes such losses as renal function, dietary and fluid restrictions, financial difficulties, uncertainty about the future, and social isolation. Research shows that over 70% of patients are unaware of their depressive symptoms and fail to recognize the need for help (Johnson and Dwyer, 2008). Therefore, understanding the intricate relationship between depression and health outcomes, including mortality in ESRD, is of paramount importance to improving patient care and quality of life.

## 5.Depression affects health outcomes in ESRD

Previous research has focused primarily on medical and physiological factors influencing health outcomes in patients with ESRD, often overlooking the significant role of mental health. However, emerging evidence suggests that the use of mental health medications is associated with improved survival rates in ESRD patients. Table 3 summarizes the key factors contributing to adverse health outcomes in this population, including impaired immune responses, heightened inflammation, malnutrition, poor adherence to treatment, and decreased physical activity.

**TABLE 3:** Factors through which depression affect health outcomes in ESRD.

Factors	Mechanism	References
Immunologic response	Increased inflammatory activation of the immune system affecting both the periphery and the central nervous system	Lee and Giuliani (2019)
Malnutrtion	Poor appetite decreases the quantity and quality of food intake	Caruana et al. (2022)
Treatment adherence	Depressive symptoms influence the treatment adherence for ESRD	Butt et al. (2022)
Inflammation response	Inflammatory cytokines affect the function of neural cells, and serve as sources of biological basis of depression	Kalender et al. (2006)
Decreased exercise	Depression impairs the muscle and physical function	Natale et al. (2019b)

Notably, patients with depression who are undergoing HD have been found to experience significantly higher rates of hospitalization (Lacson et al., 2014). van Dijk et al. (2013) found that depression is associated with both increased mortality risk and a higher prevalence of comorbidities in HD patients. Among the various risk factors affecting health outcomes in ESRD, depression is one of the most influential. Its pervasive impact underscores the need for mental health evaluation and intervention as an integral component of ESRD management.

Current guidelines for the management of ESRD emphasize the importance of assessing depression as part of routine clinical evaluations. By addressing depression alongside traditional clinical factors, healthcare providers can potentially improve overall patient outcomes, reduce hospitalizations, and enhance quality of life for individuals with ESRD.

### 5.1 Depression increases the risks of withdrawal and suicide in ESRD

In a 20-year study with 716 participants, 18.5% of all deaths were due to dialysis withdrawal (Mailloux et al., 1993). Patients who withdrew from dialysis were generally older, with 65.1% being over 61 years old. Several factors contributed to dialysis withdrawal, including cancer, catabolism, malnutrition, financial strain, and dissatisfaction with life. Notably, the rate of withdrawal was higher among white and male patients compared to Black and female patients (Nelson et al., 1994). Furthermore, older age and diabetic kidney disease were recognized as key factors affecting the choice to stop dialysis.

Alarmingly, the suicide rate among dialysis patients is 50 times that of the general population, according to Levy (2000). A comparable study utilizing the Taiwan National Health Insurance Research Database found that ESRD patients receiving HD had a notably elevated suicide risk compared to controls (Liu et al., 2017). The suicide risk was especially high in the initial 0–3 months after starting dialysis. Even with extensive national health insurance for dialysis in Taiwan, the suicide risk for ESRD patients on dialysis was still about 140% higher than that of the general population (Chen et al., 2018).

Dialysis patients use various methods of suicide, with consuming potassium-rich foods and severing HD vascular access being common (Leggat et al., 1997). A study in Taiwan found that most suicides involved severing HD vascular access. This highlights the need to address suicide risks in chronic disease patients (Shulman et al., 1989). Early diagnosis and management of depression are crucial, and healthcare providers should regularly check for suicidal thoughts in ESRD patients. If present, immediate mental health intervention is necessary. Proactive mental healthcare and strong psychosocial support can enhance quality of life and reduce mortality in these patients.

### 5.2 Depression accounts for cognitive impairment

The mechanisms behind cognitive impairment in CKD and ESRD are not well understood (Kim et al., 2022). Recent studies reveal a complex interplay between depression and cognitive impairment in ESRD, where depression can be both a symptom and a response to early cognitive decline (Chan et al., 2022; Kim et al., 2022; Tian et al., 2022; Feng et al., 2022). The main connections between depression and cognitive impairment include: 1) depression as a common symptom of cognitive dysfunction, 2) a reaction to early cognitive deficits, 3) depression’s own detrimental effects on cognitive function, and 4) its role as a risk factor or early indicator of cognitive decline. Key mechanisms linking the two include vascular injury, changes in glucocorticoid steroids, hippocampal atrophy, and increased β-amyloid plaque deposition, systemic and neuroinflammation and deficiencies in nerve growth factors or neurotrophins (Byers and Yaffe, 2011).

Vascular disease is a key link between depression and cognitive impairment, contributing to both conditions. Typically, reduced cerebral blood flow leads to cognitive issues, but CKD patients often have higher-than-normal blood flow due to anemia-induced hyperperfusion. This compensatory mechanism may temporarily offset vascular dysfunction but doesn't prevent long-term brain damage. Understanding these interactions highlights the importance of targeted treatments for CKD and ESRD patients to address these interconnected issues.

### 5.3 Depression predicts the mortality in ESRD

Kimmel et al. (2000) first showed that persistent depression increases mortality risk in HD patients, with repeated depression assessments being more reliable predictors of survival than baseline depression alone. Studies confirm depression’s link to higher death risk in dialysis patients (Burton et al., 1986), with Young et al. (2010) noting its prediction of both all-cause and CVD mortality in HD patients. While chronic depression affects ESRD mortality, the impact of depression severity is unclear. Some research links severe depression to higher hospitalization and death risks (Lopes et al., 2002; Kimmel et al., 2000). Some research links severe depression to higher hospitalization and death risks (Kimmel et al., 2000, 1998; Christensen et al., 1994).

In summary, depression significantly increases mortality risk in ESRD patients, especially through its connection to cardiovascular disease, which remains the leading cause of death in this population (van Dijk et al., 2013); Additionally, depressive disorders contribute to severe immune dysfunction, which is the second leading cause of death in ESRD (Chilcot et al., 2008). Furthermore, depression promotes inflammation, a recognized risk factor for CVD, diabetes, and various age-related diseases (Jaremka et al., 2013). Given the high prevalence of untreated depression in ESRD patients and its profound implications for survival, further research is warranted to better define the levels and specific symptoms of depression that most strongly influence mortality. Early identification and targeted interventions may help mitigate the adverse effects of depression on survival outcomes in ESRD patients.

### 5.4 Depression leads to non-adherence in ESRD

Depression is a well-recognized factor contributing to non-adherence in ESRD patients, which exacerbates mortality risks. A study showed that renal patients with depression are three times more likely to have reduced adherence to their treatment regimens compared to those without depression (Ossareh et al., 2014). This non-adherence includes failure to follow prescribed medication, dietary, fluid, and treatment regimens, all of which are essential components of dialysis prescriptions. Non-adherence directly contributes to adverse health outcomes and increased mortality rates.

Adherence to treatment is assessed using various laboratory-based and behavioral adherence indices, all of which are closely associated with patient health outcomes. For example, transplant centers often consider adherence to HD treatment as a key factor in evaluating candidates for renal transplantation. Patients with depression, however, are more likely to struggle with adherence to these regimens. Specifically, non-adherence to medications such as calcium carbonate has been associated with elevated serum phosphorus concentrations and parathyroid hormone (PTH) levels, further complicating the management of ESRD.

Effective management of depression can significantly improve medication adherence and, by extension, enhance overall patient care. Adherence to prescribed treatments is predominantly correlated with depressive symptom scores, highlighting the importance of addressing depression as part of a comprehensive care plan for ESRD patients. Early recognition and treatment of depression may mitigate the risks associated with non-adherence and improve clinical outcomes for patients with ESRD.

### 5.5 Depression predict sarcopenia in advanced CKD and dialysis

Sarcopenia, a muscle disorder characterized by reduced muscle mass, diminished quality, and impaired muscle function (e.g., strength), commonly occurs in individuals with advanced CKD or those undergoing dialysis. This condition often develops as a secondary complication of aging or the inflammatory processes related to organ failure. The progression of sarcopenia in these populations is closely linked to increased mortality and hospitalization rates (Pereira et al., 2015; Giglio et al., 2018). Notably, studies have identified a significant association between depression and sarcopenia in CKD and dialysis patients, including elderly HD patients (Kim et al., 2014) and participants in a multicenter cohort study (Kurita et al., 2021a).

The strong link between depression and sarcopenia can be explained by several factors. Depressed individuals often engage less in physical activities essential for muscle maintenance (Biddle and Asare, 2011; Heissel et al., 2023; Pearce et al., 2022). Additionally, depression is linked to higher levels of pro-inflammatory cytokines like interleukin-6, leading to chronic inflammation, protein breakdown, and muscle wasting (Chang et al., 2024; Colasanto et al., 2020; Dantzer et al., 2008). These inflammatory processes exacerbate muscle wasting and the progression of sarcopenia (Noce et al., 2021; Pan et al., 2021). Early detection and treatment of depression in CKD and dialysis patients is vital for reducing muscle loss and improving outcomes (Li et al., 2024; Liu et al., 2024b; Kurita et al., 2021b). Addressing both physical activity and inflammation may effectively alleviate sarcopenia in these patients.

## 6 Therapies for depression in ESRD

Managing depression in ESRD is complex and requires a multidisciplinary team, including nephrologists, psychiatrists, dialysis nurses, and physiotherapists. Treatment strategies are divided into non-pharmacological and pharmacological approaches.

### 6.1 Non-pharmacological treatments

Non-pharmacological treatments, such as cognitive behavioral therapy (CBT) and exercise, are vital. CBT helps restructure negative thoughts and behaviors, effectively reducing depressive symptoms in CKD patients. Exercise, in particular, is an important non-pharmacological approach for alleviating depression in ESRD patients (Mitrou et al., 2013; Grigoriou et al., 2015). It not only reduces depressive symptoms but also enhances cardiovascular health, reduces pain, and improves overall wellbeing (Grigoriou et al., 2024; Mafra and Fouque, 2014). When combined with pharmacological treatments, non-pharmacological therapies, such exercise and CBT, are essential components of a comprehensive treatment plan for depression in this population.

### 6.2 Pharmacological treatments

Pharmacological treatments are complex because renal dysfunction affects drug absorption, protein binding, and metabolism, resulting in higher drug levels. Dialysis can further reduce drug concentrations, causing potential rebound effects. These challenges complicate dosing and necessitate further research for optimization.

Tricyclic antidepressants (TCAs) and selective serotonin reuptake inhibitors (SSRIs) are commonly used to treat depression in ESRD patients. The European Renal Best Practice Guidelines recommend SSRIs as the first choice for CKD patients with moderate to severe depression (King-Wing Ma and Kam-Tao Li, 2016; Cukor and Kimmel, 2018). However, evidence for the effectiveness of SSRI is limited, with many studies lacking randomization and control. For example, a trial found fluoxetine ineffective in improving depression scores in HD patients compared to placebo (Blumenfield et al., 1997). Conversely, another study showed that 20 mg/day of fluoxetine was effective and safe for both HD patients and those with normal kidney function (Levy et al., 1996). Additionally, a trial demonstrated that the 12-week sertraline treatment significantly improved depression symptoms in 47.5% of HD patients (Taraz et al., 2013). Paroxetine combined with psychotherapy showed significant therapeutic effects, leading to improvements in depressive symptoms and increases in several nutritional markers in HD patients (Koo et al., 2005). Wuerth et al. also studied antidepressant treatment in 44 ESRD patients on peritoneal dialysis (PD). They found that sertraline, citalopram, bupropion, nefazodone, or paroxetine led to significant improvements in depressive symptomatology in 23 patients who completed the treatment (Wuerth et al., 2003). Table 4 summarizes the commonly used antidepressants and their recommended dosages for ESRD patients. Table 5 provides an overview of various studies evaluating SSRIs for depression in ESRD patients, highlighting the mixed results and the need for more controlled trials. Although antidepressants show positive results, their safety and efficacy in dialysis patients remain unclear. A systematic review on SSRIs in these patients was inconclusive due to the limited number of studies and short follow-up periods (Palmer et al., 2016). More clinical trials are necessary to determine the best antidepressant therapy for dialysis patients.

**TABLE 4:** Treatments for depression in patients with ESRD.

Treatment	References
Psychotherapy	Natale et al. (2019b)
Electroconvulsive therapy	McGrane et al. (2022)
Selective serotonin and norepinephrine reuptake inhibitors	Nguyen et al. (2019), Scirica et al. (2019)
Selective serotonin reuptake inhibitors	Vangala et al. (2020)
Herbal supplements	Cao et al. (2019)
Acupressure	Suandika et al. (2023), Kim et al. (2016)
Other antidepressants: Bupropion, maprotiline, mirtazapine	Cohen et al. (2007)

**TABLE 5:** SSRI for depression in patients with ESRD.

Drug	Subject	Dose	Therapeutic effect	Reference
Fluoxetine	14 HD patients with depression	20 mg/d	−	Blumenfield et al. (1997)
Fluoxetine	7 HD patients with depression; 9 depressed patients with normal renal function	20 mg/d	+	Levy et al. (1996)
Sertraline	50 HD patients with depression	50 mg/d (the first 2 weeks); 100 mg/d (other 10 weeks)	+	Taraz et al. (2013)
Paroxetine+ psychotherapy	62 HD patients	10 mg/d	+	Koo et al. (2005)
Sertraline/citalopram/bupropion/nefazodone/paroxetine	44 PD patients with depression		+	Wuerth et al. (2003)

PD, Peritoneal dialysis; HD, Hemodialysis.

Vitamin D deficiency is a potential depression risk factor in ESRD patients, and some studies suggest that vitamin D supplementation might help boost serotonin and modulating inflammation (Somoza-Moncada et al., 2023; Jahan-Mihan et al., 2024). Depression and suicide are both linked to low serotonin levels and systemic inflammation (Grudet et al., 2014). However, a trial with 746 dialysis patients in Southeast China found that high-dose vitamin D3 did not significantly alleviate depressive symptoms in those with vitamin D deficiency (Wang et al., 2016). This highlights the need for more research to understand how vitamin D contributes to depression management in ESRD patients.

### 6.3 Challenges in antidepressant use for ESRD patients

Treating depression in ESRD patients is challenging due to side effects like arrhythmias and dry mouth, which result from polypharmacy and altered drug metabolism, requiring careful dose adjustments. Physical activity is crucial for reducing depressive symptoms and offers benefits like improved cardiovascular function and enhanced cerebral blood flow. Treatment should be guided by specialists using a multidisciplinary approach involving nephrologists, psychiatrists, social workers, and dialysis staff. More research is needed on safer antidepressants for ESRD patients, as there is currently a lack of clinical trials. Moving forward, integrating suitable medication with regular physical exercise should be encouraged as a comprehensive strategy for managing depression in ESRD. In summary, treating depression in ESRD involves careful management of both medications and non-pharmacological therapies. Antidepressants should be personalized based on kidney function, while also incorporating physical activity and possibly vitamin D. Caution is necessary due to altered drug metabolism and potential side effects. More research is needed to improve treatment and outcomes.

## 7 Nanomaterials for the treatment of depression

Current antidepressants can cause significant side effects and interactions, underscoring the need for new treatments. Nanoparticle-based drug delivery systems show promise in improving drug delivery across the blood-brain barrier, controlling release, and altering pharmacokinetics (van den Broek et al., 2022; Tosi et al., 2020). They can target specific sites and safely deliver drugs, offering a potential solution for challenging brain diseases. Table 6 provides a summary of nanomaterials used in depression treatment. Further research is needed to assess the efficacy of these treatments for ESRD.

**TABLE 6:** Nanomaterials for the treatment of depression.

Nanomaterials	Constitution	Mechanism	Reference
Polyphenol-armored nanomedicine	TNF-α-siRNA, a GAGQD–encapsulated BSA nanoparticle (siRNA-GBSA NP) core, and CHI/TA multilayer shell	Targeted modulation of gut microbiota-brain interactions	He et al. (2023)
Plant polyphenols and their nano-formulations	Berberine, piperine, curcumin, naringenin, ascorbic acid and ginsenosides	Differentiation and inhibition of neuronal cell apoptosis, promotion of neuronal cell survival and modulation of key neurotransmitters	Kabra et al. (2022)
Curcumin-based nanoformulations	Various curcumin nanoformulations	Increased bioavailability of nanoformulated curcumin compared to free curcumin	Ataei et al. (2023)
BDNF-quercetin alginate nanogels	Quercetin-based alginate nanogels (quercetin nanogels) loaded with BDNF (BDNF-quercetin nanogels) composed of thermosensitive gel	Regulation of the glutamatergic system, PI3K-Akt, and BDNF-TrkB signaling pathway	Xu et al. (2023)
Exosome-sheathed ROS-responsive nanogel	PACAP and estrogen (E2), sheathed with exosomes	Antioxidant and anti-inflammatory properties and may regulate the expression of pivotal proteins in the PACAP/PAC1 pathway to promote synaptic plasticity	Hu et al. (2023)
Melanin-like PDA NPs	PDA NPs were prepared by the oxidative polymerization of dopamine hydrochloride (DA-HCL)	Anti-inflammatory and rescuing synaptic loss	Zhu et al. (2023)
Nickel oxide nanoparticles (NiO NPs)	NiO NPs originated from the Cressa nudicaulis plant extract. The integrated NiO NPs were loaded with doxepin drug	Sufficiently long targeted-release properties	Lu et al. (2023)

BSA, Bovine serum albumin; GAGOD, Gallic acid–mediated graphene quantum dot; PACAP, Pituitary adenylate cyclase-activating polypeptide; BDNF, Brain-derived neurotrophic factor; CHI/TA, Chitosan and tannin acid; PDA NPs, polydopamine nanoparticles.

Oral drug administration for depression is common but has drawbacks, such as first-pass metabolism and adherence issues, leading to variable plasma levels and side effects (Andrade, 2019). Intranasal delivery offers a promising alternative by bypassing the blood-brain barrier and first-pass metabolism, thereby reducing side effects (Alberto et al., 2022). Future psychiatric treatments may increasingly rely on non-oral methods, such as implants and transdermal systems. These options offer the potential for more regulated and prolonged drug release, with fewer side effects, thereby enhancing the overall therapeutic effectiveness for conditions such as depression.

## 8 Traditional Chinese medicine (TCM) in the treatment of depression in ESRD

Rencently, there has been a significant focus on exploring herbal remedies from Traditional Chinese Medicine (TCM) as possible treatments for depression. Certain TCM formulas have shown great effectiveness, targeting multiple areas with minimal toxicity. These formulas make promising choices for treating depression, even though the exact molecular mechanisms are not yet understood (Table 7). In TCM, depression is classified as an “emotional disease,” whith depressive symptoms historically described in various terms such as “epilepsy,” “insomnia,” “hysteria,” “lily disease,” and “sensation of gas rushing” in ancient Chinese medical texts. Based on the emotional mutual-restriction theory, the idea of “joy counteracting sadness” was employed to improve low spirits. Furthermore, ancient Chinese texts, such as the *Shen Nong Ben Cao Jing*, recommended the use of herbs like “pipistrelle” and “albizzia” to help restore both physical function and emotional balance. Although these treatments show promise, depression remains a major challenge in modern medicine due to the incomplete understanding of its pathogenesis. Currently, no existing treatments can completely stop or reverse the early progression of depression.

**TABLE 7:** Traditional Chinese medicine treatment for depression in ESRD.

Traditional Chinese medicine	Subject	Dose	References
Suanzaoren soup	68 MHD with depression	1 dose/day	Wang (2018)
Chai Hu Shu Gan San	80 MHD with depression and sleep disorder	1 dose/day	Fang et al. (2020)
Bushen-Shugan	88 MHD with anxiety and depression	1 dose/day	Hu and Wang (2020)
Tiaoxue Shugan soup	150 MHD with depression	1 dose/day	Yu (2019)
Meigui Jieyu soup	88 MHD with depression	1 dose/day	Zhao et al. (2017)
Xiaofeng Zhiyang Granule	8 HD with skin itch	3 bags per time,and 2 times per day	Ren (2022)
Zhenyuan Capsule	57 MHD with anxiety, depression, and chronic heart failure	2 capsules per time,and 3 times per day	Liu et al. (2019)

PD, Peritoneal dialysis; HD, Hemodialysis; MHD, Maintenance Hemodialysis.

## 9 Special population

In recent decades, the aging global population has contributed to a rise in chronic diseases among the elderly, with older adults showing the highest rates of CKD. Depression, linked to higher mortality and reduced quality of life, increases their vulnerability (Farrokhi et al., 2014). However, studies on elderly ESRD patients are limited. Research by Alencar et al. (2020) found high depression rates in older HD patients, which correlated with lower quality of life, reduced serum albumin, and higher parathyroid hormone levels. This suggests that assessing depression and quality of life should be key components in managing these patients.

Another significant concern is the notably elevated mortality rates in women under the age of 45. Within these populations, various female hormonal disorders can result in menstrual irregularities, anovulation, infertility, sexual abnormalities, early menopause, accelerated bone loss, and a potentially heightened risk of cardiovascular complications (Guglielmi, 2013). Sexual disorders are prevalent in this demographic, often correlating with depression and a decline in quality of life. Khaira et al. (2012) conducted a study examining depression and marital dissatisfaction among Indian HD patients and their spouses. The study found that male spouses experienced greater marital stress than their female counterparts. Despite the high prevalence of depression among female patients, it remains underdiagnosed and undertreated. It is well-established that women generally have a longer life expectancy than men. Therefore, gender-specific medical and psychosocial conditions significantly impact female patients with ESRD, warranting increased attention and tailored care.

## 10 Conclusion and prospection

Depression is common in ESRD patients and is linked to higher mortality and worsening kidney function. Poor kidney function is associated with a higher likelihood of depression. Early and accurate depression assessment is vital for improving patients’ quality of life. Factors such as malnutrition, pain, sleep disorders, cardiovascular disease, and unemployment can trigger depression in ESRD patients. Depression worsens health outcomes, raising the risk of treatment withdrawal, suicide, and cognitive issues. This study is the first to highlight the significant benefits of nanomaterials and traditional Chinese medicine in treating depression, with minimizing side effects. It's important to focus on specific groups, especially older adults and women under 45, to enhance intervention strategies.

Nonetheless, there are still several obstacles: 1) To confirm these associations, thorough randomized controlled trials need to be designed and conducted. 2) Additionally, patients should be formally screened when starting HD treatment. 3) A thorough examination of the risk factors for depression in ESRD is urgently required. 4) Future treatments for depression in ESRD should prioritize the development of medications with minimal side effects.
